# Supplementary material for: From sequence to enzyme mechanism using multi-label machine learning
Source: BMC Bioinformatics. 2014 May 19;15:150. doi: 10.1186/1471-2105-15-150 (PMC4229970; doi:10.1186/1471-2105-15-150)
Supplement: Additional file 2 — Java code of ml2db. Additional file ml2db_code.tar.gz contains the Java source code to run the multi-label machine learning experiments and save the results to database. The code’s Javadoc is included. [file 1471-2105-15-150-S2.zip › additional file 2/ml2db/ml2db/doc/main/ml2db/mulan/attributesfiltered/AttFiltArff.html]

AttFiltArff


JavaScript is disabled on your browser.


- Overview
- Package
- Class
- Use
- Tree
- Deprecated
- Index
- Help

- Prev Class
- Next Class

- Frames
- No Frames

- All Classes

- Summary:
- Nested |
- Field |
- Constr |
- Method

- Detail:
- Field |
- Constr |
- Method


main.ml2db.mulan.attributesfiltered

## Class AttFiltArff

- java.lang.Object
- - uk.ac.standrews.utils.main.Initialised
  - - main.ml2db.weka.Arff
    - - main.ml2db.mulan.mulanarff.MulanArff
      - - main.ml2db.mulan.attributesfiltered.AttFiltArff

- All Implemented Interfaces:
  :   uk.ac.standrews.utils.main.Initialisable

  ---

    

  ```
  public class AttFiltArff
  extends MulanArff
  ```

- - ### Field Summary

    - ### Fields inherited from class main.ml2db.mulan.mulanarff.MulanArff

      `m_massiveFile`
    - ### Fields inherited from class uk.ac.standrews.utils.main.Initialised

      `m_initialisationIsCorrect`
  - ### Constructor Summary

    Constructors

    Constructor and Description

    `AttFiltArff(java.lang.String dataPropsFileName,
    DbManagers dbMan,
    AttFilter filterManager,
    int oldArffRecordId)`
  - ### Method Summary

    Methods

    | Modifier and Type | Method and Description |
    | --- | --- |
    | `java.util.TreeSet<java.lang.String>` | `getAttributesNames()` |
    | `static java.lang.String` | `getFilteredArffName(int oldArffId, int oldTestId, int oldTrainId)` |
    | `AttFilter` | `getFilterManager()` |

    - ### Methods inherited from class main.ml2db.mulan.mulanarff.MulanArff

      `deriveXmlPath, generateXmlFile, getDataSetManager, getInstances, getInstancesNames, getXMLpathFromArffPath, isMassiveFile, main, run, run, setDataSetManager`
    - ### Methods inherited from class main.ml2db.weka.Arff

      `checkAndInitialise, fillArff, generateAndWriteArffFile, getArffPath, getArffRecordId, getDataArffProperties, getDataDbManager, getDataPropsFilePath, getDbConn, getDbManagers, getHeaderString, getInputDbReader, getInstancesString, getResultsDbManager, setArffRecordId, setup, writeArff`
    - ### Methods inherited from class uk.ac.standrews.utils.main.Initialised

      `initialisationIsCorrect, setInitialisation, setInitialisationIsCorrect, setInitialisationNotCorrect`
    - ### Methods inherited from class java.lang.Object

      `equals, getClass, hashCode, notify, notifyAll, toString, wait, wait, wait`

- - ### Constructor Detail


    - #### AttFiltArff

      ```
      public AttFiltArff(java.lang.String dataPropsFileName,
                 DbManagers dbMan,
                 AttFilter filterManager,
                 int oldArffRecordId)
      ```
  - ### Method Detail


    - #### getAttributesNames

      ```
      public java.util.TreeSet<java.lang.String> getAttributesNames()
      ```


    - #### getFilterManager

      ```
      public AttFilter getFilterManager()
      ```


    - #### getFilteredArffName

      ```
      public static java.lang.String getFilteredArffName(int oldArffId,
                                         int oldTestId,
                                         int oldTrainId)
      ```


- Overview
- Package
- Class
- Use
- Tree
- Deprecated
- Index
- Help

- Prev Class
- Next Class

- Frames
- No Frames

- All Classes

- Summary:
- Nested |
- Field |
- Constr |
- Method

- Detail:
- Field |
- Constr |
- Method
